# Genetic Diagnosis of Non-Syndromic Hearing Loss in South Indian Consanguineous Families Using Whole-Exome Sequencing

**DOI:** 10.3390/medicina62061040

**Published:** 2026-05-28

**Authors:** Jayakumar Swetha, Yogesh Vetriselvan, Manoranjani Murugan, Irisappan Ganesh, Sambandam Ravikumar, Kumar Rangarajalu, M. Manju, Ballambattu Vishnu Bhat

**Affiliations:** 1 Department of Biochemistry, Aarupadai Veedu Medical College and Hospital, Vinayaka Mission’s Research Foundation (DU), Kirumampakkam, Puducherry 607403, India; swetha.jayakumar@avmc.edu.in (J.S.); drmanjumay1@gmail.com (M.M.); 2 Department of Medical Biotechnology, Faculty of Interdisciplinary Studies, Aarupadai Veedu Medical College and Hospital, Vinayaka Mission’s Research Foundation (DU), Kirumampakkam, Puducherry 607403, India; yogeshbioinfo04@gmail.com (Y.V.); manobiotech95@gmail.com (M.M.); ganesh.irisappan@avmc.edu.in (I.G.); ravikumar.sambandam@avmc.edu.in (S.R.); 3 Advisor Medical Research and Publications, Aarupadai Veedu Medical College and Hospital, Vinayaka Mission’s Research Foundation (DU), Kirumampakkam, Puducherry 607403, India

**Keywords:** non-syndromic hearing loss, genetic heterogeneity, genetic testing, homozygosity mapping, *MYO15A*

## Abstract

*Background and Objectives*: Hereditary hearing loss is the most common auditory disability among various disabilities. Consanguineous populations have been found to have autosomal recessive disorders twice as often as in the general population. This study aimed to highlight the phenotypic and genetic complexity of non-syndromic hearing loss (NSHL) in South Indian consanguineous families. *Materials and Methods*: Whole-exome sequencing (WES) was performed on individuals with NSHL who were negative for common deafness-causing genes (*GJB2*, *GJB6*, *SLC26A4*, and *MTRNR1*). The candidate variants identified were correlated with ROH regions identified using the Automap tool. Sanger sequencing was performed for validation, followed by segregation analysis for the available family members. The effects of the candidate variants were analyzed using an in silico structural approach and the ACMG guidelines. *Results:* WES identified variants, including a stop-gain, an indel, and a missense mutation, in the genes *SIX1*, *MYO7A*, *MYO3A*, and *MYO15A*. Three variants were classified as likely pathogenic, one variant as a variant of uncertain significance (VUS), and one variant as likely benign. Homozygous variants in *MYO15A* and *MYO7A* were identified within ROH regions, indicating autosomal recessive inheritance. Additionally, two heterozygous variants in the *SIX1* and *MYO3A* genes were identified. This study indicates a high degree of genotypic and phenotypic heterogeneity of hearing loss among affected individuals. *Conclusions*: This integrated approach, which combines homozygosity mapping with WES, could be effective for diagnosing NSHL in affected individuals. Further genetic screening and characterization of NSHL in consanguineous families is also warranted. Genetic testing in high-risk populations could be a valuable method for diagnosing genetic hearing loss in children.

## 1. Introduction

Hearing loss is a common sensory disability that affects millions of people worldwide [1]. It is a complex disorder with a variety of causes, including environmental causes, perinatal infections, genetic factors, and certain medical conditions [2]. More than 50% of cases of congenital hearing loss are due to genetic factors. Hereditary hearing loss (HHL) is classified as syndromic or non-syndromic and primarily affects the inner ear, causing sensorineural HL [2,3]. The clinical severity ranges from mild to profound, with different frequencies (low, mid, and high), and is often non-progressive [4]. Non-syndromic hearing loss (NSHL) follows both autosomal and sex-linked patterns of inheritance, and 70% of cases are due to autosomal recessive inheritance patterns [3,5].

Syndromic hearing loss is often readily diagnosed due to characteristic extra-auditory clinical features that facilitate identification of the underlying etiology [6]. In contrast, NSHL lacks associated systemic manifestations and exhibits considerable clinical heterogeneity in terms of age of onset, severity, and laterality, making etiological determination more challenging [5,7]. Hence, comprehensive genetic analysis is required to elucidate the underlying genetic cause in many cases of NSHL.

Consanguineous marriages increase the likelihood of identity-by-descent and homozygosity for rare deleterious variants, thereby elevating the prevalence of recessive disorders [8]. In India, particularly in South Indian ethnicities where consanguinity remains culturally prevalent, this practice contributes to a higher burden of recessive conditions, including NSHL [9,10,11]. Despite this increased genetic risk, the mutational spectrum of NSHL children in the South Indian consanguineous population remains incompletely characterized.

*GJB2*, *GJB6*, *SLC26A4*, *MTRNR1*, and *CDH23* are common deafness-causing genes predominantly found in Asia. Targeted single-gene screening is unable to address the genetic heterogeneity of NSHL in this population [10,12]. Hence, WES was conducted, providing a comprehensive approach to identify both known and novel pathogenic variants across multiple HL-associated genes [13]. This integrated approach, combining homozygosity mapping with WES, may be effective for improving molecular diagnosis of NSHL in affected individuals, particularly in a consanguineous population. Thus, the current study helps in understanding the genetic basis of NSHL offspring born to consanguineous parents in South Indian consanguineous families.

## 2. Materials and Methods

### 2.1. Subjects and Clinical Evaluations

**Figure 1:**
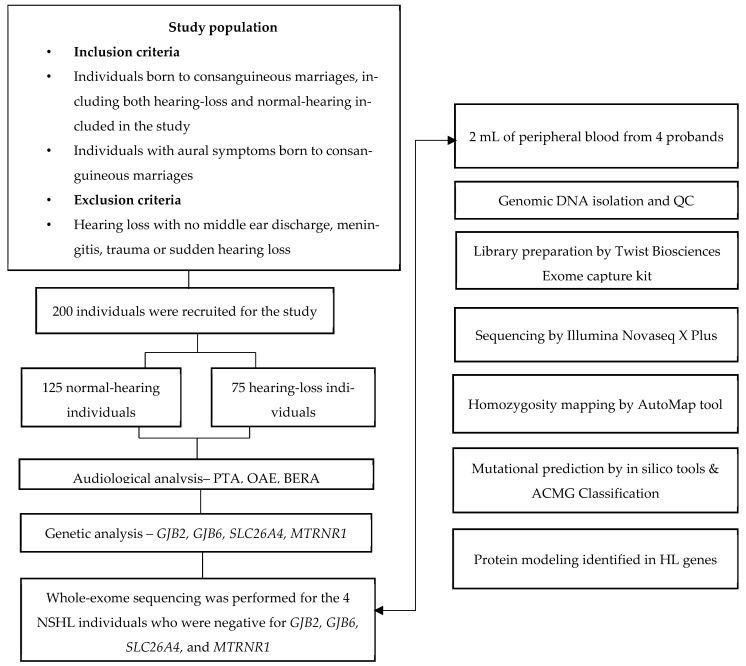
Workflow of the study.

A total of 200 unrelated individuals with NSHL and normal-hearing individuals born to consanguineous parents were recruited for the study (Figure 1) from the ear, nose, and throat (ENT) unit of Aarupadai Veedu Medical College and Hospital, Puducherry, India. Within this cohort, we performed WES on 4 NSHL patients who were negative for common deafness-causing genes, including *GJB2*, *GJB6*, *SLC26A4*, and *MTRNR1*. Probands were born to consanguineous parents, and the degree of consanguinity is also mentioned in Table 1. The ethics committee approved the study, and all participants provided informed consent. Medical histories were collected using detailed questionnaires, and audiological and ophthalmological examinations were performed. Clinical history was obtained to assess symptoms suggestive of vestibular dysfunction. Impedance testing was performed to assess middle ear function, and pure-tone audiometry (PTA) was conducted to assess air- and bone-conduction thresholds at 250 Hz, 500 Hz, 1000 Hz, 2000 Hz, 4000 Hz, and 8000 Hz. No other systemic clinical abnormalities were reported in the patients.

### 2.2. Genetic Testing

After analyzing the common deafness genes, no pathogenic variants were observed in the 4 probands affected with NSHL. WES was performed on the probands to identify the genetic etiology of hearing loss. Genomic DNA was extracted from peripheral blood using the QIAamp mini kit (Qiagen, Hilden, Germany) according to the manufacturer’s instructions. Exome libraries were prepared using the Twist Human Core Exome kit (South San Francisco, CA, USA), and paired-end sequencing (150 bp) was performed on the Illumina NovaSeq X Plus platform (Illumina, San Diego, CA, USA), achieving an average sequencing depth of approximately 100× across targeted regions. Raw sequencing reads were aligned to the human reference genome (GRCh38/hg38) using the Burrows–Wheeler Aligner (BWA-MEM, v0.7.10). Duplicate reads that were mapped to multiple genomic locations were removed before downstream analysis. To reduce alignment artifacts, local realignment around insertion–deletion (InDels) regions was performed. Variant calling for single-nucleotide variants (SNVs) and small InDels was conducted using the Genome Analysis Toolkit (GATK, version 4). The resulting variants were annotated using the wANNOVAR pipeline. Autozygosity mapping was performed to detect runs of homozygosity (ROH) from WES data. The Automap tool (https://automap.iob.ch/, accessed on 30 October 2025) identifies ROH regions and variants from WES data [14]. Rare variants were identified by filtering variants with a minor allele frequency (MAF) ≤ 0.005% based on population frequency data from the gnomAD, ExAC, and 1000G. Novel variants were further annotated using wANNOVAR [15]. Hearing-loss-associated variants were shortlisted using multiple parameters, including gene functional annotation, the HL-associated gene list from the HHL homepage (https://hereditaryhearingloss.org/ accessed on 25 December 2025), and the available literature. Clinically relevant mutations in both coding and non-coding regions were identified using databases such as ClinVar, OMIM, HGMD, LOVD, and DECIPHER. All presumably non-disturbing protein variants (introns, UTRs, intergenic regions, synonymous variants, etc.) were filtered out. Only nonsynonymous and splice-site variants were used for clinical interpretation. Candidate variants were confirmed by Sanger sequencing. PCR amplification was conducted using the primers listed in Supplementary Table S1, followed by ExoSap-IT (Affymetrix Inc., Santa Clara, CA, USA) clean-up. Purified PCR products were sequenced using the ABI Prism BigDye Terminator cycle sequencing Ready Reaction kit v. 3.1 (Applied Biosystems, Foster, CA, USA). Sequences were determined using the SeqStudio Genetic Analyzer (Applied Biosystems, Thermo Fisher Scientific, Woonsocket, RI, USA) and analyzed using the SeqScape^®^ Software version 3 (Applied Biosystems, Thermo Fisher Scientific, Waltham, MA, USA).

### 2.3. Variant Interpretation

Functional prediction and pathogenicity assessment of identified variants were performed using in silico tools, including SIFT (https://siftdna.org/www/SIFT_seq_submit2.html, accessed on 10 January 2026) FATHMM (http://fathmm.biocompute.org.uk/, accessed on 10 January 2026), PolyPhen-2 (http://genetics.bwh.harvard.edu/pph2/, accessed on 11 January 2026), Mutation Taster (http://www.mutationtaster.org/, accessed on 11 January 2026), Mutation Assessor (https://github.com/sanderlab/MutationAssessor_r4, accessed on 10 January 2026), Mutpred2 (https://github.com/vpejaver/mutpred2, accessed on 10 January 2026), CADD (https://cadd.gs.washington.edu/snv, accessed on 11 January 2026), GERP, and REVEL. Finally, variants were classified as benign, likely pathogenic, or variants of uncertain significance (VUSs) in accordance with the guidelines of the American College of Medical Genetics and Genomics (ACMG) [16,17].

### 2.4. In Silico Structural Prediction

Additionally, structural modeling was performed to understand the impact of the identified variants. The three-dimensional structures of the SIX1 (Q15475), MYO15A (Q9UKN7), and MYO7A (Q13402) proteins were retrieved from the UniProt database, and the wild-type and mutant structures of the proteins were modeled using the Swiss Model server. The modeled structures were then evaluated using the SAVES v6.0 server, and the Ramachandran plot was used to assess structural stereochemical properties. The structures were further visualized using PyMOL v.3.12 software. The Root Mean Square Deviation (RMSD) values quantified structural deviations. RMSD < 1 Å indicated minimal conformational change; 1–2 Å suggested moderate structural alteration.

## 3. Results

**Figure 2:**
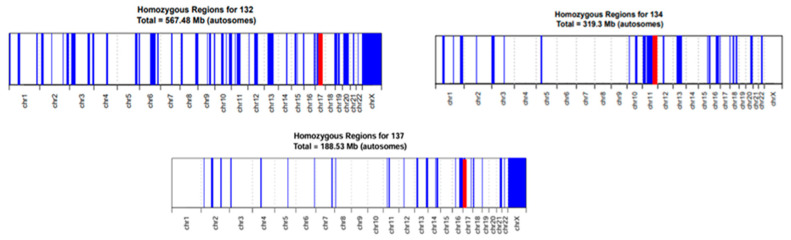
Homozygosity mapping using the Automap tool.

In this study, WES identified that all four probands were affected with NSHL consistently with South Indian consanguineous origins, and their clinical characteristics are listed in Table 1. The probands showed no dysmorphic features or clinical deformities. The identified variants were listed in Table 2. ROH analysis using the Automap tool was performed for the probands with homozygous variants, as shown in Figure 2, and the autozygous regions in probands carrying homozygous variants, as shown in Supplementary Tables S2–S4. All probands exhibited high ROH regions distributed across all chromosomes, consistent with a high degree of genomic autozygosity due to parental consanguinity. The homozygous regions of each proband included 567.48 Mb in Family 132, 319.3 Mb in Family 134, and 188.53 Mb in Family 137. Furthermore, these ROH regions likely contributed to the homozygous state of pathogenic variants identified in the respective probands, facilitating variant prioritization in the WES analysis.

The proband HL33, a 17-year-old male, presented with congenital bilateral NSHL with moderate-to-profound hearing loss at high frequencies. Clinical evaluation revealed a history of hearing impairment in the father; however, molecular testing was not available for confirmation of segregation. At the time of evaluation, the proband had no other clinical conditions. WES identified a heterozygous in-frame deletion, c.397_399del (p.Glu133del), in the *SIX1* gene in the HL33 proband, which is associated with DFNA23 (OMIM 601205). This variant has already been reported in BOR syndrome. The *SIX1* gene is a developmental gene that plays a major role in mammalian development, especially during organogenesis. The gene located at chromosome 14q23.1 encodes a SIX homeoprotein [18].

**Figure 3:**
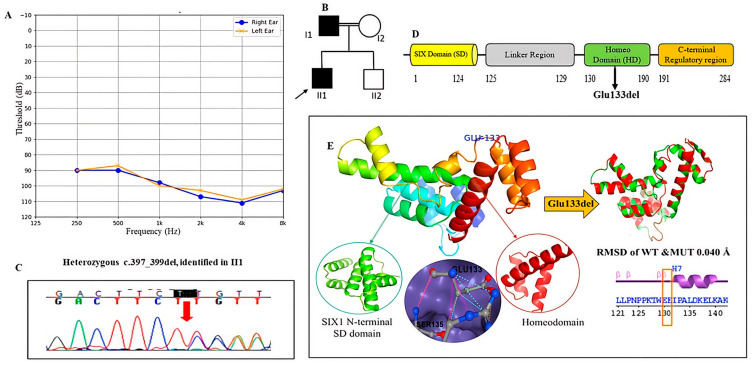
(**A**) Audiogram showing bilateral profound hearing loss in proband II1. Pedigree of the family is indicated in (**B**). Sanger validation of the variant is shown in (**C**). Gene structure of SIX with the variant mentioned in (**D**). A comparison of wild and mutant structures of the SIX1 protein in (**E**). ↗ indicates the proband, ▀ and indicates the hearing-loss patient.

The heterozygous mutation c.397_399del (p. Glu133del) in the *SIX1* gene has already been reported in the ClinVar database. The variant was validated using Sanger sequencing, as mentioned in Figure 3. Furthermore, this variant was found to have an allele frequency of 0.000001239 in the gnomAD database, indicating that it is rare and hence PM2. Based on previously reported functional evidence, PS3_Supporting and PP1 were applied [18,19]. Since this in-frame deletion affects a conserved region of the protein, it meets PM4 and PM1 criteria. By applying these attributes—PM2, PP1, PS3_Supporting, PM1, and PM1—the effect of this in-frame variant present in exon 1 is found to be likely pathogenic according to the ACMG guidelines. A comparison of wild-type and mutant structural predictions showed an RMSD of 0.004, consistent with the conserved domains of the SIX1 protein in Figure 3.

**Figure 4:**
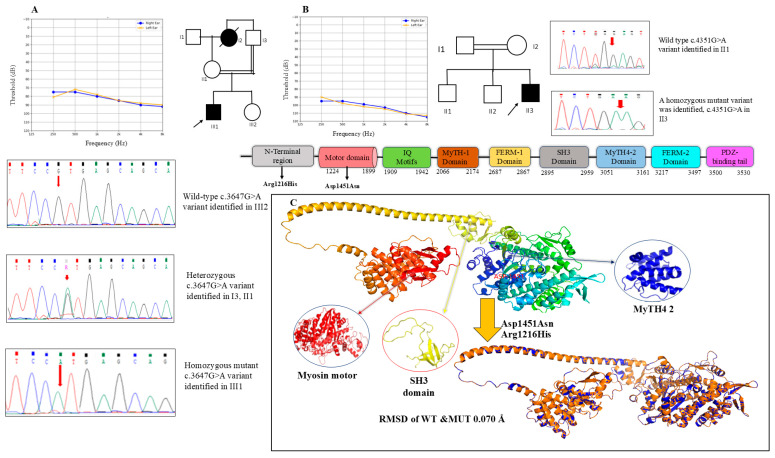
(**A**) Audiogram showing NSHL in proband III1. Pedigree of the family and segregation analysis of family HL132. (**B**) Audiogram showing NSHL in proband II3. Pedigree of the family and segregation analysis of family HL134. Gene structure of MYO15A with the variant mentioned in (**C**).

The proband HL132, a 17-year-old male, presented with congenital bilateral NSHL with moderately severe NSHL with sloping at high frequencies. The proband, who was born to parents with an uncle–niece relationship (I3, II1), and the proband’s grandmother had congenital bilateral sensorineural hearing loss. In contrast, the proband’s mother has normal hearing thresholds in both ears. Further, the proband’s father and younger sister have normal hearing. WES identified a homozygous missense variant, c.3647G>A p. Arg1216His in the *MYO15A* gene, which encodes for the protein myosin XVa. It is a member of the unconventional myosin superfamily. It plays an important role in the graded elongation of stereocilia and in actin organization in hair cells of the inner ear, which are essential for normal-hearing function [20]. A mutation in the *MYO15A* gene is responsible for autosomal recessive NSHL (DFNB3, OMIM 600316). The c.3647 G>A variant was identified in the N-terminal domain of MYO15A [21]. The missense c.3647G>A (p. Arg1216His) variant in the *MYO15A* gene was identified in the homozygous state in the affected proband. Segregation analysis revealed that both the father and mother were heterozygous for the variant, whereas the proband’s younger sister was wild-type, consistent with an autosomal recessive pattern of inheritance, as shown in Figure 4A. The variant frequency in population databases is relatively high; hence, BS1 was applied. PP1 was applied, since the variant is segregated within the family, and PM1_S is due to a moderate functional domain level. Additionally, computational predictions indicate a moderate effect providing supporting evidence (PP3). Hence, this variant is considered likely benign.

Additionally, we identified a heterozygous missense variant, c.2651C>G (p.Ser884Cys), in the *MYO3A* gene. This variant has a very low allele frequency in population databases (0.00002214), supporting PM2_Supporting. In silico prediction tools consistently suggest deleterious effects, supporting PP3. Hence, based on these criteria, PM2 + PP3, the variant is classified as a variant of uncertain significance (VUS).

The proband HL134, a 10-year-old male, presented with prelingual, profound NSHL. At the age of 3, the proband underwent a cochlear implant. Clinical evaluation revealed no other syndromic features and normal vestibular function. WES identified a homozygous missense variant, c.4351G>A p. Asp1451Asn in the *MYO15A* gene, in the proband HL134. This c.4351G>A (p. Asp1451Asn) variant was already classified as likely pathogenic. The variant was confirmed by Sanger sequencing, as shown in Figure 4B. The proband’s first elder brother was found to be wild-type for this variant; segregation analysis was not performed in full due to the unavailability of family members’ samples.

The allele frequency of this variant in population databases (0.000007434) supports PM2. The variant is located in a functionally important region of the motor domain of the MYO15A protein, supporting PM1 (moderate). PM3 was applied in its homozygous state in the proband, highly consistent with the MYO15A-related autosomal recessive NSHL phenotype. In silico prediction tools have shown deleterious effects, which support PP3. Hence, this variant is classified as likely pathogenic. Furthermore, the in silico structural predictions for both of the variants present in the N-terminal domain and the motor domain of MYO15A (p. Arg1216His and p. Asp1451Asn) are mentioned in Figure 4C, with a 0.070 Å minimal RMSD value indicating a structure rearrangement.

**Figure 5:**
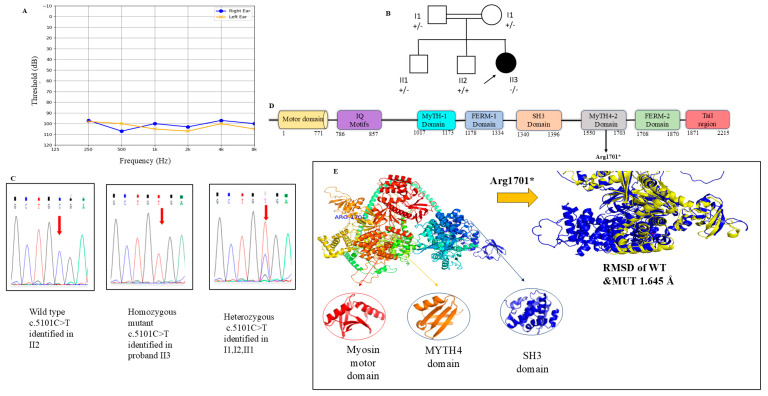
(**A**) Audiogram showing bilateral profound hearing loss in proband II3. Pedigree of the family indicated in (**B**). Sanger validation and segregation analysis of the variant is shown in (**C**). Gene structure of MYO7A with variant mentioned in (**D**). A comparison of wild and mutant structures of MYO7A protein in (**E**). (+/+ denotes wild type, +/− denotes heterozygote, −/− denotes homozygous mutant. ↗ indicates the proband, ● hearing loss patient.

The HL137 proband, a 17-year-old female born to third-degree consanguineous parents, presented with prelingual, profound bilateral NNHL. Vestibular function was normal in the proband, with no other clinical abnormalities at the time of evaluation. In the HL137 proband, WES identified a homozygous stop-gained mutation, c.5101C>T (p.Arg1701*), in the *MYO7A* gene, which is associated with DFNB2 and DFNA11 (OMIM 601205). This variant has already been reported in Usher’s syndrome type 2B [22]. The validation for this variant is shown in Figure 5. Segregation analysis revealed that both unaffected parents were carriers of the variant; the first elder brother had a heterozygous variant, and the second elder brother had a wild-type variant, which is consistent with an autosomal recessive pattern of inheritance. Mutations in *MYO7A* have been associated with three disorders: dominant and recessive NSHL (DFNA11 and DFNB2, respectively) and Usher’s syndrome type 1B [23]. In this study, the proband had no ophthalmic dysfunction. Further follow-up is recommended for this proband to identify late-onset clinical manifestations. The *MYO7A* gene encodes the actin-based motor protein myosin-VIIa, which is especially crucial for the function of cochlear hair cells and eye development [22,24].

According to the ACMG/AMP guidelines, the c.5101C>T (p. Arg1701*) stop-gained variant in the *MYO7A* gene was classified as likely pathogenic. As a null variant predicted to result in loss of function in a gene where loss of function is a known disease mechanism, it meets PVS1. Segregation analysis within the family provides supporting evidence (PP1). Furthermore, this variant was found to have an allele frequency in the gnomAD database, indicating that it is rare and hence PM2_Supporting. In silico structural prediction analysis showed an RMSD of 1.645 Å, which also characterizes conserved domains of the MYO7A protein shown in Figure 5.

## 4. Discussion

This study expands the current understanding of the molecular basis of NSHL in South Indian consanguineous populations using WES. Consanguineous marriages increase genetic homozygosity via identity-by-descent, and they remain a significant risk factor for autosomal recessive disorders [8]. In our study, four unrelated HI probands from South Indian consanguineous families who were negative for common deafness genes (*GJB2*, *GJB6*, *SLC26A4,* and *MTRNR1*) underwent exome sequencing to elucidate the etiologies of HL. Four variants were identified in three HL genes, highlighting the genetic heterogeneity of NSHL in this study. Our findings emphasize the role of well-established hearing-loss-associated genes, including *MYO15A*, *MYO7A*, *SIX1*, and *MYO3A*, in consanguineous families of South Indian origin.

In family HL33, the c.397_399del (p. Glu133del) variant in the *SIX1* gene was identified in the proband. Previous reports have shown that this variant exhibits clinical heterogeneity ranging from bilateral NSHL to branchiootic (BO) syndrome and branchio-oto-renal (BOR) syndrome [18,25]. Table 3 explains the genotype–phenotype correlation of the variant (Glu133del). This variant is found in the homeodomain of the *SIX1* gene, underscoring its functional significance. According to the ACMG guidelines, this variant is classified as pathogenic or likely pathogenic.

The *SIX1* gene encodes a homeodomain transcription factor. SIX1 has two highly conserved domains, namely, the SIX domain (SD), which specifically binds with transcriptional co-activators of the EYA family (such as EYA1 and EYA2), and homeodomains (HD), which have direct DNA-binding capacity [26]. The interaction of the EYA-SIX-PAX complex helps regulate embryonic development of the ear, branchial arches, and kidneys, particularly inner ear neurosensory cell differentiation, maintenance of normal cell proliferation, and differentiation of cochlear neurons in the spiral ganglia [19,26].

The study by Salam et al., in which families initially classified with autosomal dominant NSHL were later reevaluated and diagnosed with the BOR spectrum, highlighted the phenotypic variability of *SIX1* [18]. Similarly, the proband in family HL33 presented with NSHL, which can mimic non-syndromic hearing loss and may manifest subclinical features over time. Given the incomplete penetrance of *SIX-related* gene disorders, longitudinal follow-up with renal evaluation and detailed clinical screening is recommended to detect late-onset manifestations.

In the present study, we identified two homozygous missense variants in the *MYO15A* gene associated with NSHL in two unrelated consanguineous families. In family HL132, the homozygous variant c.3647G>A (p.Arg1216His) was detected along with heterozygous c.2651C>G (p.Ser884Cys) in the *MYO3A* gene. In family HL137, the variant c.4351G>A (p.Asp1451Asn) had already been reported in India. The recurrence of homozygous variants in the *MYO15A* gene in a consanguineous population provides strong genetic evidence of their pathogenic role in HL, with an AR pattern [27].

*MYO15A* encodes myosin XVa, a key protein involved in stereocilia elongation and actin organization in cochlear hair cells, processes that are essential for normal mechanotransduction [24]. Both variants are in the motor domain, suggesting protein function disruption as the likely disease mechanism. The genotype–phenotype correlation across the two families highlights the critical contribution of *MYO15A* to congenital sensorineural hearing loss. This underscores the value of MYO15A domain genetic testing for accurate molecular diagnosis and genetic counseling in consanguineous populations.

Additionally, a heterozygous missense variant in the *MYO3A* gene is expressed in the mammalian retina and inner ear [22]. Variants in this gene are known to cause autosomal recessive NSHL (DFNB2), autosomal dominant hearing loss (DFNA11), and Usher’s syndrome type 1B [28,29]. However, further investigation is recommended to confirm the role of *MYO3A* in the proband.

In family HL137, WES identified a homozygous stop-gained variant c.5101C>T (p.Arg1701*) in *MYO7A*, a gene encoding the actin-based motor protein myosin-VIIa, which is essential for cochlear hair cell function and retinal integrity [22]. Variants in this gene are known to cause autosomal recessive NSHL (DFNB2), autosomal dominant hearing loss (DFNA11), and Usher’s syndrome type 1B [22,23,30]. The detected variant introduces a premature termination codon, consistent with a loss-of-function mechanism, and has previously been reported in individuals with Usher’s syndrome [23]. According to the ACMG criteria, it is classified as likely pathogenic. Clinically, no ophthalmic abnormalities were observed; however, because biallelic truncating variants are frequently associated with syndromic presentations, long-term ophthalmologic monitoring is recommended to detect potential late-onset retinitis pigmentosa. Overall, the findings support *MYO7A*-associated recessive hearing loss in this family, with important implications for genetic counseling, surveillance for syndromic features, and consideration of cochlear implantation for auditory rehabilitation.

Clinical heterogeneity in the mutation spectrum of deafness genes may vary among different ethnic groups, particularly in consanguineous populations [12]. Targeted screening of common deafness genes may miss rare or population-specific variants in genetically heterogeneous NSHL; hence, WES could be an effective tool for molecular diagnosis [25]. Although a small sample size limits the present study, it demonstrates the role of genetic testing for NSHL diagnosis. Continued clinical follow-up combined with detailed phenotypic correlation may further clarify genotype–phenotype relationships, particularly in cases where NSHL phenotypes overlap with syndromic presentations.

## 5. Conclusions

The current study highlights the role of WES in the genetic diagnosis of NSHL in the South Indian consanguineous population. The identification of pathogenic and likely pathogenic variants in key deafness-associated genes highlights the genetic heterogeneity underlying NSHL in this population. Early genetic diagnosis can facilitate timely interventions, such as auditory rehabilitation, and guide surveillance for potential syndromic manifestations in NSHL-mimicking genes with variable expressivity. Integrating genomic testing into routine clinical evaluation in high-risk consanguineous communities may significantly improve diagnostic yield, enable risk assessment for families, and support preventive strategies through awareness and counseling.

## Figures and Tables

**Table 1:** Clinical evaluation of probands in the study.

S. No	Family ID	Age/Sex	DOC	Type of HL & Severity	Onset	Method of Hearing Rehabilitation	Family History, If Any
1.	HL33	17/M	3°	Profound SNHL	Prelingual	No	Yes
2.	HL132	17/M	2°	Moderately severe SNHL	Prelingual	No	Yes
3.	HL134	10/M	3°	Profound SNHL	Prelingual	Cochlear implant	No
4.	HL137	17/F	3°	Profound SNHL	Prelingual	No	Yes

DOC—degree of consanguinity (2°—second degree of consanguinity, 3°—third degree of consanguinity).

**Table 2:** Variants identified from WES in this study.

S. No	Family ID	Genes	HGVS	Genotype	Type	Allele Frequency	SIFT	Polyphen2	Mutation Taster	ACMG Attributes	Classification
1.	HL33	*SIX1*	NM_005982.4 c.397_399del, (p. Glu133del)	Het	In-frame deletion	0.000001239	-	-	Deleterious	PM2+PM4, PM1, PS3_supporting	Likely pathogenic
2.	HL132	*MYO15A*	NM_016239.4 c.3647G>A (p. Arg1216His)	Hom	Missense	0.0006832	Tolerated	Probably damaging	Deleterious	BS1+PP1, PM1_S+PP3	Likely Benign
		*MY03A*	NM_017433.5 c.2651C>G p.Ser884Cys	Het	Missense	0.00002214	Damaging	Probably damaging	Disease causing	PM2+PP3	VUS
3.	HL134	*MYO15A*	NM_016239.4 c.4351G>A (p. Asp1451Asn)	Hom	Missense	0.000007434	Damaging	Probably damaging	Deleterious	PM1+PM2+PM3+PP3	Likely pathogenic
4.	HL137	*MYO7A*	NM_000260.4 c.5101C>T (p. Arg1701*)	Hom	Stop-gain	0.000003820	-	-	Deleterious	PVS1+PM2_S+PP1	Likely pathogenic

Hom—Homozygous; Het—Heterozygous.

**Table 3:** Genotype–phenotype correlation of the SIX1 Glu133del variant.

Study/Reference	Family ID	Sex/Age	Phenotype	Onset of HL	Syndromic Features	ACMG
Present study	HL33	17/M	SNHL	Prelingual	None	Likely Pathogenic
Lee et al. [19]	SH613-1214	11/F	SNHL	Postlingual	None	Likely Pathogenic
Salam et al. [18]	F1120	NA	SNHL	NA	Branchial defects, solitary left hypodysplastic kidney with vesico-ureteral reflux and progressive renal failure (BOR syndrome)	Pathogenic

## Data Availability

The primary data supporting the findings of this paper are available on request.

## Supplementary Materials

The following supporting information can be downloaded at https://www.mdpi.com/article/10.3390/medicina62061040/s1: Table S1: Primers used in the study, Table S2: Proband in Family 132, Table S3: Proband in Family 134, Table S4: Proband in Family 137.
