# Supplementary material for: Genetic Diagnosis of Non-Syndromic Hearing Loss in South Indian Consanguineous Families Using Whole-Exome Sequencing
Source: Medicina (Kaunas). 2026 May 28;62(6):1040. doi: 10.3390/medicina62061040 (PMC13304154; doi:10.3390/medicina62061040)
Supplement: Supplementary file 1 [file medicina-62-01040-s001.zip › medicina-4226024-supplementary.pdf]

Supplementary Table S1: Primers used in the study

Gene	Exon	Forward primer (5'–3')	Reverse primer (5'-3')	Amplicon
SIX1	1	CTGCCGTCGTTTGGCTTTAC	TCTCTTTGCCTCCGGTTCTT	527
MYO15A	3	CTGAAGAAGAGGCCACCCTG	CTTGGCTTGGGGTCCTGAAT	409
MYO15A	12	CACCACACTACTGGTCAGGG	AGGATGCGGAAGATGCTGTC	206
MYO7A	37	ACGGCATCAATGAGAGGACC	TGGGGGCCTGAAGTAGTCAT	342

Supplementary Table S2: Proband in Family 132

#Chr	Begin	End	Size(Mb)	Nb_variants	Percentage_homozygosity
chr1	2611214	11020333	8.41	194	97.42
chr1	69148684	84866125	15.72	67	92.54
chr1	2.22E+08	2.31E+08	9.23	175	97.71
chr2	14232607	17816758	3.58	39	92.31
chr2	17817064	30787111	12.97	219	96.35
chr2	95150272	97142612	1.99	27	92.59
chr2	1.87E+08	1.9E+08	4	43	88.37
chr2	2.2E+08	2.27E+08	7.66	70	97.14
chr2	2.3E+08	2.38E+08	7.53	138	98.55
chr3	12967314	45500591	32.53	270	95.93
chr3	1.49E+08	1.68E+08	18.66	114	92.98
chr3	1.87E+08	1.98E+08	10.46	142	93.66
chr4	1.01E+08	1.15E+08	13.63	97	91.75
chr5	1.46E+08	1.63E+08	17.72	217	98.62
chr5	1.74E+08	1.81E+08	7	154	98.05
chr6	89613905	1.3E+08	39.99	249	93.98
chr6	1.49E+08	1.54E+08	5.18	69	92.75
chr7	88335827	93994146	5.66	32	100
chr8	8381430	10697586	2.32	29	96.55
chr8	10924662	17077725	6.15	67	92.54
chr8	1.25E+08	1.45E+08	19.86	346	97.69
chr9	76503715	77728671	1.22	30	90
chr9	91356005	1.05E+08	14.12	174	93.68
chr9	1.3E+08	1.32E+08	2.16	82	96.34
chr9	1.35E+08	1.38E+08	3.43	202	99.5
chr10	51699238	67811578	16.11	58	93.1
chr10	93629367	95606989	1.98	31	93.55
chr10	1.17E+08	1.22E+08	5.26	63	95.24
chr10	1.28E+08	1.34E+08	5.58	124	96.77
chr11	3373664	6199939	2.83	108	92.59
chr11	33108451	34992320	1.88	36	88.89
chr11	44267521	56700288	12.43	137	97.08
chr11	56701244	72233179	15.53	481	97.92
chr12	6072469	9753471	3.68	159	96.23
chr12	54532087	79199290	24.67	259	93.05
chr13	26682591	74764967	48.08	273	95.6
chr14	65032745	72892397	7.86	64	90.62
chr15	24681039	32158627	7.48	71	94.37
chr15	32168218	39584211	7.42	88	97.73
chr15	44560235	45605573	1.05	41	92.68
chr15	92171937	1.02E+08	9.55	79	97.47
chr16	82858432	90075265	7.22	297	96.97
chr17	4179925	6426597	2.25	104	98.08
chr17	14236248	21298407	7.06	113	90.27
chr17	26301794	36171845	9.87	130	93.85
chr17	36254620	49312652	13.06	493	96.75

chr18	74129654	80133156	6	64	93.75
chr19	282753	8888697	8.61	592	98.48
chr19	8888884	16762129	7.87	379	96.04
chr19	23654393	35359120	11.7	112	94.64
chr20	7906378	19280979	11.37	91	95.6
chr20	22036380	51607436	29.57	385	95.06
chr21	24587674	32317982	7.73	48	91.67
chr22	15528179	18126279	2.6	33	93.94
chrX	284193	1345233	1.06	42	100
chrX	2691262	1.37E+08	134.18	485	91.96
chrX	1.37E+08	1.56E+08	18.9	121	98.35

INFO: 567.48 Mb are in Homozygous Regions (autosomal chromosomes)
AutoMap v1.0 used for analysis
Variant filtering parameters used: DP=8, percaltlow=.25, percalthigh=.75, binomial=.000001, maxgap=10
Other parameters used: window=7, windowthres=5, minsize=1, minvar=25, minperc=88, chrX=Yes, extend=1

Supplementary Table S3: Proband in Family 134

#Chr	Begin	End	Size(Mb)	Nb_variants	Percentage_homozygosity
chr1	56707388	75806969	19.1	150	92.67
chr1	1.57E+08	1.59E+08	1.45	34	88.24
chr1	2.16E+08	2.42E+08	25.86	395	95.44
chr2	1.08E+08	1.1E+08	1.86	26	88.46
chr3	1278587	10046582	8.77	72	95.83
chr3	10087281	23967788	13.88	129	96.12
chr3	1.11E+08	1.12E+08	1.04	26	88.46
chr5	41927321	52909113	10.98	39	89.74
chr10	20211814	26513704	6.3	41	92.68
chr10	74082394	90772675	16.69	137	97.08
chr11	6877527	28023872	21.15	304	93.42
chr11	42321522	56700311	14.38	153	96.08
chr11	56701244	96129413	39.43	686	96.94
chr12	43432474	51452306	8.02	135	94.81
chr13	33165290	73845863	40.68	201	91.04
chr15	81359310	85579423	4.22	52	90.38
chr15	94314436	1.02E+08	7.41	66	100
chr16	50217679	70120577	19.9	232	95.26
chr16	70156498	74409891	4.25	72	93.06
chr16	74417986	77884511	3.47	59	98.31
chr16	85735111	87888048	2.15	35	88.57
chr18	166710	2718267	2.55	45	91.11
chr18	26460000	36111013	9.65	87	88.51
chr18	50392269	62539755	12.15	119	95.8
chr20	41098360	56370727	15.27	165	96.97
chr22	26373498	35067256	8.69	137	94.16

INFO: 319.3 Mb are in Homozygous Regions (autosomal chromosomes)

AutoMap v1.0 used for analysis
Variant filtering parameters used: DP=8, percaltlow=.25, percalthigh=.75, binomial=.000001, maxgap=10
Other parameters used: window=7, windowthres=5, minsize=1, minvar=25, minperc=88, chrX=Yes, extend=1

Supplementary Table S4: Proband in Family 137

#Chr	Begin	End	Size(Mb)	Nb_variants	Percentage_homozygosity
chr2	24119662	26309623	2.19	31	93.55
chr2	85754638	97162093	11.41	79	93.67
chr2	97167846	1.05E+08	7.92	80	95
chr2	1.67E+08	1.72E+08	5.21	99	93.94
chr3	11028856	16493722	5.46	77	98.7
chr4	68551772	75940706	7.39	120	93.33
chr5	1.11E+08	1.15E+08	4.01	31	90.32
chr6	1.59E+08	1.6E+08	1.26	29	96.55
chr7	1.38E+08	1.48E+08	9.45	156	92.95
chr8	10924545	12133373	1.21	37	94.59
chr11	33864748	34947442	1.08	33	93.94
chr11	47425795	56375649	8.95	82	89.02
chr12	39640897	42119015	2.48	34	88.24
chr13	19177404	21157889	1.98	30	90
chr13	21176522	27963105	6.79	90	92.22
chr13	98018387	1.14E+08	16.22	203	97.54
chr14	65084098	67557417	2.47	29	89.66
chr14	75135079	87963572	12.83	78	93.59
chr16	28497126	31915298	3.42	91	88.04
chr16	58670957	70130345	11.46	88	90.91
chr16	70535518	74409891	3.87	75	93.33
chr16	74413226	90075069	15.66	380	98.42
chr17	137587	2335447	2.2	94	98.94
chr17	9925431	18483854	8.56	157	96.18
chr17	18492630	21298407	2.81	54	88.89
chr17	68600322	72849810	4.25	44	90.91
chr18	5434196	10455080	5.02	36	100
chr18	10797569	12308274	1.51	30	93.33
chr19	327998	1785773	1.46	180	97.78
chr21	31120886	46662002	15.54	351	96.58
chr22	23573465	28030140	4.46	80	96.25
chrX	2714441	1.36E+08	133.52	522	92.53
chrX	1.39E+08	1.56E+08	17.05	92	94.57

INFO: 188.53 Mb are in Homozygous Regions (autosomal chromosomes)
AutoMap v1.0 used for analysis
Variant filtering parameters used: DP=8, percaltlow=.25, percalthigh=.75, binomial=.000001, maxgap=10
Other parameters used: window=7, windowthres=5, minsize=1, minvar=25, minperc=88, chrX=Yes, extend=1
